# The Efficacy of Anti-Tumor Necrosis Factor Alpha for Symptomatic Stricturing Small Bowel Crohn’s Disease

**DOI:** 10.7759/cureus.10315

**Published:** 2020-09-08

**Authors:** Mansour Alourfi, Mahmoud Mosli, Omar I Saadah

**Affiliations:** 1 Internal Medicine, King Faisal Medical City for Southern Regions, Abha, SAU; 2 Internal Medicine, King Abdulaziz University, Jeddah, SAU; 3 Gastroenterology, King Abdulaziz University Hospital, Jeddah, SAU; 4 Inflammatory Bowel Disease Research Group, King Abdulaziz University, Jeddah, SAU; 5 Pediatrics/Gastroenterology, King Abdulaziz University Hospital, Jeddah, SAU

**Keywords:** anti-tnf-α, stricture, adalimumab, infliximab, surgery, saudi arabia, crohn’s disease

## Abstract

Introduction

Crohn’s disease (CD) is a chronic inflammatory disease. Current treatment aims to prevent complications and the need for surgical intervention. In patients with symptomatic complications, such as strictures, the possible benefits of anti-tumor necrosis factor-alpha (anti-TNF-α) therapy are currently the subject of considerable debate. This study aims to determine whether anti-TNF-α therapy could decrease the need for or delay the time until surgery in patients with CD presenting with symptomatic strictures of the small bowel in the King Abdulaziz University Hospital (KAUH), Saudi Arabia.

Methods

We conducted a retrospective, single-center study that assessed the need for surgical treatment in adult patients with symptomatic stricturing CD who were treated conventionally or with TNF-α inhibitors. Simple logistic regression was used to examine the association between surgical resection and biologics therapy and stepwise elimination logistic regression analysis was used to identify predictors of surgical resection.

Results

In total, 75 patients fulfilled the study criteria with 50 in the anti-TNF-α arm and 25 in the conventional arm. Surgical resection was required for six patients (12.2%) in the anti-TNF-α treatment arm and one patient (4%) in the conventional treatment arm (P=0.26). Endoscopic balloon dilatation was performed in two patients (4%) in the anti-TNF-α arm and one patient (4%) in the conventional arm (P=0.69). No statistically significant association was observed between surgical resection and treatment with biologic therapy (odds ratio [OR]=0.50, 95% CI: 0.16-1.53, P=0.22). Stepwise elimination identified age (OR=4.54, 95% CI: 0.79-25.11, P=0.09) and disease duration (OR=1.01, 95% CI: 1.00-1.02, P=0.004) as significant predictors of surgery.

Conclusions

In this cohort, anti-TNF-α therapy did not provide additional benefits with regards to avoiding or delaying surgery in CD patients with stricturing of the small bowel.

## Introduction

Crohn’s disease (CD) is a subtype of chronic inflammatory bowel disease (IBD) that can affect any part of the gastrointestinal tract. Most patients present with a predominantly inflammatory phenotype at the time of diagnosis. Even though the therapeutic options for CD have increased, up to 70% of patients still develop complications within 10 years of diagnosis [1]. Complications such as strictures and fistulas can lead to bowel obstructions that generally require surgical intervention. Strictures affect more than 30% of patients and are considered a major cause of morbidity and hospitalization [2]. Biological therapies, including TNF-α inhibitors, have been proven to be effective for inflammatory and fistulizing CD [3,4], such as the anti-TNF-α antibodies adalimumab (ADL) and infliximab (IFX), which have similar efficacies [5]. However, the use of anti-TNF-α therapy as a treatment for CD patients with already established strictures is controversial. Some studies have suggested that it does not lead to better outcomes [6,7], while other studies have indicated a favorable therapeutic effect in this context [8-12]. Recent data from a CREOLE study has shown that the majority of CD patients with symptomatic bowel strictures responded successfully to ADL treatment [13]. Due to the lack of sufficient evidence and individual risk stratification tools favoring this approach, patients with bowel strictures are alternatively managed by surgical resection [14]; therefore, more studies are needed to evaluate the use of anti-TNF-α therapy in this patient group.

This retrospective study aimed to determine whether anti-TNF-α therapy could decrease the need for or delay the time until surgery in patients with CD presenting with symptomatic strictures of the small bowel.

## Materials and methods

Participants and study design

This retrospective single-center study included adult patients (>18 years of age) with CD and radiologically or endoscopically confirmed stricturing small bowel disease registered in the King Abdulaziz University Hospital (KAUH) inflammatory bowel disease information system (IBDIS) database between January 2008 and December 2019. KAUH, a 1067-bed hospital in Jeddah, represents the largest tertiary care hospital in the Western region of the Kingdom of Saudi Arabia. A small bowel (jejunum or ileum) stricture was defined as a constant luminal narrowing documented on endoscopic or radiological assessment or during surgery with pre-stenotic dilation or obstructive signs. A symptomatic stricture was defined as “recurrent abdominal pain, vomiting, abdominal distension, and constipation in the presence of a confirmed small bowel stricture”. Patients treated with anti-TNF-α therapy (ADL or IFX) for at least 12 weeks were included in the anti-TNF-α treatment arm; patients treated with conventional therapy (5-aminosalicylic acid [5-ASA], azathioprine [AZA], or methotrexate [MTX]) were included in the conventional treatment arm (comparator). CD diagnosis was based on common clinical, endoscopic, histological, and radiological diagnostic criteria for the disease. Patients with non-resolving complete bowel obstruction at baseline were excluded from the analysis. Data on demographics (age, sex, and smoking status), disease characteristics (location, severity, and perianal disease status), symptoms, endoscopic activity, biochemical markers (complete blood count, albumin, and C-reactive protein [CRP]), and radiological assessments (computed tomography findings and magnetic resonance enterography [MRE] findings) were collected.

Study outcomes

The primary outcome of the study was to evaluate the need for surgical resection during the follow-up period. The secondary outcomes were to uncover the rate of interventional endoscopic balloon dilatation of the stricture and the duration in months until surgical resection, if required.

Statistical analysis

All data were entered in an Excel database (Excel 2007; Microsoft Corp., Redmond, WA, USA) and analyzed using STATA 11.2 (StataCorp, College Station, Texas, USA). Baseline means and the corresponding standard deviations (SD) were calculated for continuous variables, while frequencies and percentages were calculated for categorical variables. Simple logistic regression was used to examine the association between surgical resection and biologics therapy and stepwise elimination logistic regression analysis was used to identify predictors of surgical resection. Precision of point estimates was presented using 95% confidence intervals (CIs). Significance level was set at P=0.05.

Ethical statement

The participants’ data were kept confidential, and study approval was granted by the Ethics Committee at KAUH (Ref. number I42-18) prior to the commencement of data collection.

## Results

Baseline characteristics

The IBDIS registry included 643 patients with IBD, of whom 348 had CD. After applying the inclusion and exclusion criteria, 75 patients were included in the final analysis with 50 in the anti-TNF-α treatment arm and 25 in the conventional treatment arm. Males comprised 64% of the cohort in the treatment arm and 60% in the conventional treatment arm. In total, 90% of patients in the treatment arm and 75% of patients in the conventional treatment arm were between 17 and 40 years of age (A2 according to the Montreal classification). The mean duration of symptoms was 103.7±69.0 months for the treatment arm and 108.0±66.5 months for the anti-TNF-α treatment arm; the mean disease duration was 98.6±70.80 months for the treatment arm and 106.0±67.10 months for the conventional treatment arm. The ileocolonic phenotype of the disease was observed in 72% of the anti-TNF-α group and 48% of the conventional treatment group, while the isolated ileal phenotype was found in 26% of patients treated with anti-TNF-α and in 48% of patients who received conventional treatment. Twenty-four percent of the anti-TNF-α group and 16% of the conventional treatment group had perianal disease. Extraintestinal manifestations were found in 27% of the anti-TNF-α treatment group and in 18% of the conventional treatment group. Obstructive gastrointestinal symptoms were reported by 81% of both patient groups, and 99% of them reported using corticosteroids at least once during the disease course. Concomitantly used therapy in both arms included 5-amino salicylic acid (5-ASA) derivatives, azathioprine (AZA), and methotrexate (MTX). In the treatment arm, 84% of patients had no previous surgery and only 8% had undergone previous surgery. In contrast, the conventional treatment arm had significantly fewer patients with no surgical history prior to the study (60%) whilst 36% had undergone surgery once and 4% had undergone more than one surgery (Table 1).

**Table 1:** Baseline characteristics of the 75 patients included in the study. *Chi square or t-test **P<0.05

	Total cohort (n=75) N (%) or mean±SD	Anti-TNF-α treatment (n=75) N (%) or mean±SD	Conventional treatment (n=25) N (%) or mean±SD	*P-value
Demographics				
Gender				
Females	28 (37%)	18 (36%)	10 (40%)	0.74
Males	47 (63%)	32 (64%)	15 (60%)	
Smoking Status				
Never	58 (78%)	41 (82%)	17 (68%)	0.95
Current	9 (12%)	6 (12%)	3 (12%)	
Past	4 (5%)	3 (6%)	1 (4%)	
Disease Characteristics				
Duration of symptoms (months)	104.9 ± 67.80	103.7 ± 69.00	108 ± 66.50	0.83
Duration of disease (months)	100.9 ± 69.20	98.6 ± 70.80	106 ± 67.10	0.68
Previous surgeries				
Never	57 (76%)	42 (84%)	15 (60%)	0.01**
One surgery	13 (16%)	4 (8%)	9 (36%)	
>1 surgery	5 (8%)	4 (8%)	1 (4%)	
Montreal Classification				
Age category				
A2 (17–40 years of age)	64 (85%)	45 (90%)	19 (76%)	0.11
A3 (above 40 years of age)	11 (15%)	5 (10%)	6 (24%)	
Disease location				
L1: Ileal location	25 (33%)	13 (26%)	12 (48%)	0.12
L2: Colonic location	2 (3%)	1 (2%)	1 (4%)	
L3: Ileo-colonic location	48 (64%)	36 (72%)	12 (48%)	
Perianal disease (P)	16 (21%)	12 (24%)	4 (16%)	0.43
Disease course				
Frequent relapse (>1/year)	18/72 (25%)	11/49 (22%)	7/22 (32%)	0.25
Chronic active (no remission)	5/72 (7%)	5/49 (10%)	0/22 (0%)	
Infrequent relapse (<1/year)	48/72 (67%)	33/49 (67%)	15/22 (68%)	
Extra intestinal manifestations				
Yes	17/71 (24%)	13/49 (27%)	4/22 (18%)	0.45
No	54/71 (76%)	36/49 (73%)	18/22 (82%)	
Concomitant medications				
5-aminosalicylic acid	27/73 (37%)	18/49 (37%)	9/24 (38%)	0.95
Azathioprine	53/75 (71%)	37/50 (74%)	16/25 (64%)	0.37
Methotrexate	1/75 (1%)	0/50 (0%)	1/25 (4%)	0.16
Laboratory investigations				
Hemoglobin (g/dL)	12.04±1.80	12.10±1.80	11.95±10.90	0.79
C-reactive protein (mg/dl)	33.90±42.09	33.43±20.60	34.73±18.10	0.90
Albumin (g/L)	32.14±7.07	33.04±6.19	30.36±26.80	0.13

Study outcomes

Following stricture diagnosis, surgical resection was required for seven patients (9.3%). The reason for surgical intervention was intestinal obstruction in four patients (57%) and refractory symptoms in three patients (43%). Surgical resection was performed in six patients (12.2%) in the anti-TNF-α treatment arm and one patient (4%) in the conventional treatment arm (P=0.26). Endoscopic balloon dilatation was performed in two patients (4%) in the anti-TNF-α treatment arm and one patient (4%) in the conventional treatment arm (P=0.69) (Table 2).

**Table 2:** Treatment outcomes

Outcomes	Total N=75	Anti-TNF-α treatment group N=50	Conventional treatment group N=25	P-value
Surgical resection				
Yes	7 (9%)	6 (12%)	1 (4%)	0.26
No	68 (91%)	44 (88%)	24 (96%)	
Endoscopic dilatation				
Once	1 (1%)	1 (2%)	0 (0%)	0.69
More than once	2 (3%)	1 (2%)	1 (4%)	
None	72 (96%)	48 (96%)	25 (96%)	

We evaluated the association between surgical resection and biologic therapy using simple logistic regression, and no statistically significant association was observed (OR=0.50, 95% CI 0.16-1.53, P=0.224) (Table 3).

**Table 3:** Logistic regression analysis for surgical resection adjusting for duration of symptoms. CI, confidence interval; OR, odds ratio.

Predictor	OR	P-value	95% CI
Treatment group (N=50)	0.50	0.224	0.16–1.53
Duration of symptoms (months)	1.01	0.003	1.00–1.02

Furthermore, stepwise elimination identified age (OR=4.54, 95% CI: 0.79-25.11, P=0.09) and disease duration (OR=1.01, 95% CI: 1.00-1.02, P=0.004) as significant predictors of surgery (Table 4).

**Table 4:** Predictors of surgical resection using according to stepwise elimination analysis. CI, confidence interval; OR, odds ratio.

Predictor	OR	P-value	95% CI
Age	4.54	0.09	0.79–25.11
Duration of symptoms (months)	1.01	0.004	1.00–1.02

## Discussion

Despite the advancements in available therapies for CD, the morbidity and mortality associated with the disease remains high. It is estimated that 80% of patients will require surgical intervention within 20 years of active disease. Stricture formation is the most common complication of CD and is generally accompanied by symptoms of obstruction that require hospitalization and surgery. However, surgical resection of strictures is not curative, and patients often need recurrent surgical interventions over the course of the disease [15,16]. In recent years, the need for hospitalization and surgery for patients with CD has been declining. In a population-based study by Kaplan et al., the observed decline was 7.4% between 1997 and 2009 [17]. However, it has been suggested that the decline in the need for surgery cannot be attributed to the use of anti-TNF-α therapies [3,18] and the four-fold increase in costs for the biological treatment has not been translated into savings in inpatient care [19]. In this cohort of patients with confirmed stricturing ileal CD, the overall surgical rate was 9.3% (7/75), which is in line with recent findings [13].

In inflammatory and fistulizing CD, the use of anti-TNF-α agents is recommended because therapies can prevent complications [3,4]; a similar efficacy is observed with both ADL treatment and IFX treatment [5]. However, a number of studies suggest that patients with a non-stricturing CD phenotype that initially respond to anti-TNF-α therapy also report significant rates of intestinal strictures and obstructions [20]. A prospective study by Condino et al., which evaluated 36 patients with CD, found that obstructive symptoms could develop in up to 10% of patients with CD treated with anti-TNF-α therapy [21]. However, limited and controversial evidence exists on the use of anti-TNF-α therapy in CD patients presenting with stricturing complications. Therefore, in this study, we retrospectively evaluated the use of ADL and IFX in symptomatic stricturing CD. Overall, surgical resection was required in 9.3% of patients over the course of the disease (101±69 months), while 16% of patients had surgery at least once prior to the start of the study and an additional 8% had repeated surgery. We did not observe any statistically significant relationships that prevented or delayed the need for surgery in patients treated with ADL or IFX in comparison with conventional therapy. In fact, we noted that more patients in the anti-TNF-α treatment group (12%) required surgical resection compared to patients in the conventional treatment group (4%). However, this difference was not statistically significant and may have been caused by selection bias (84% of patients had not undergone previous surgery in the anti-TNF-α treatment arm in comparison with 60% in the conventional treatment arm, P=0.01).

A recent prospective CREOLE study, which evaluated the efficacy of ADL in 97 patients with symptomatic bowel strictures, reported that patients in the treatment arm benefited from ADL therapy (64% of patients at week 24). They also reported that the presence of a total stricture length of <12 cm, intermediate dilation of the small bowel proximal to the stricture, and the absence of fistulae predicted treatment success [13]. Moreover, the study suggested that anti-TNF-α therapy is effective for inflammatory strictures but not for fibrotic strictures, a distinction that can be difficult to establish in clinical practice. Results from a multicenter retrospective study by Bamba et al. suggested that anti-TNF-α therapy should be considered in patients with a short stricture length to help avoid intestinal resection [8]. However, it is important to note that it is difficult to compare results between previous studies because of varying study designs and non-uniform classification of strictures and symptoms; therefore, these findings should be analyzed with care.

The main limitations of our study include the small sample size and retrospective, single-center design. Therefore, a larger, prospective study is needed to evaluate the benefits of anti-TNF-α therapy in patients with CD that already present with stricturing disease.

## Conclusions

Our findings reveal that anti-TNF therapy did not provide additional benefit in terms of avoiding or delaying the need for surgery in this cohort of Saudi patients with symptomatic stricturing ileal Crohn's disease.
